# Mike Cashel: magic spot magician

**DOI:** 10.1128/jb.00588-25

**Published:** 2026-02-25

**Authors:** Deborah M. Hinton, Susan Gottesman

**Affiliations:** 1 Gene Expression and Regulation Section, Laboratory of Biochemistry and Genetics, National Institute of Diabetes and Digestive and Kidney Diseases, National Institutes of Health 2511, Bethesda, Maryland, USA; 2 Laboratory of Molecular Biology, Center for Cancer Research, National Cancer Institute 3421 , Bethesda, Maryland, USA; Dartmouth College Geisel School of Medicine, Hanover, New Hampshire, USA

**Keywords:** ppGpp, RelA, SpoT, RNA polymerase

## EDITORIAL

In July 2025, we lost Mike Cashel, a gifted scientist and critical contributor to the bacterial research community, after a long illness. Through his studies on (p)ppGpp and its modes of action, Mike had a major influence on our understanding of bacterial physiology and how bacteria respond to changes in nutrient availability. It would be hard to overstate his groundbreaking contributions to understanding how cells deal with the loss of nutrients, a fundamental question in biology. Mike was a generous mentor and colleague, and we at the NIH as well as his friends around the world already miss his wisdom, insight, and deep understanding of bacterial physiology.

**Fig 1:**
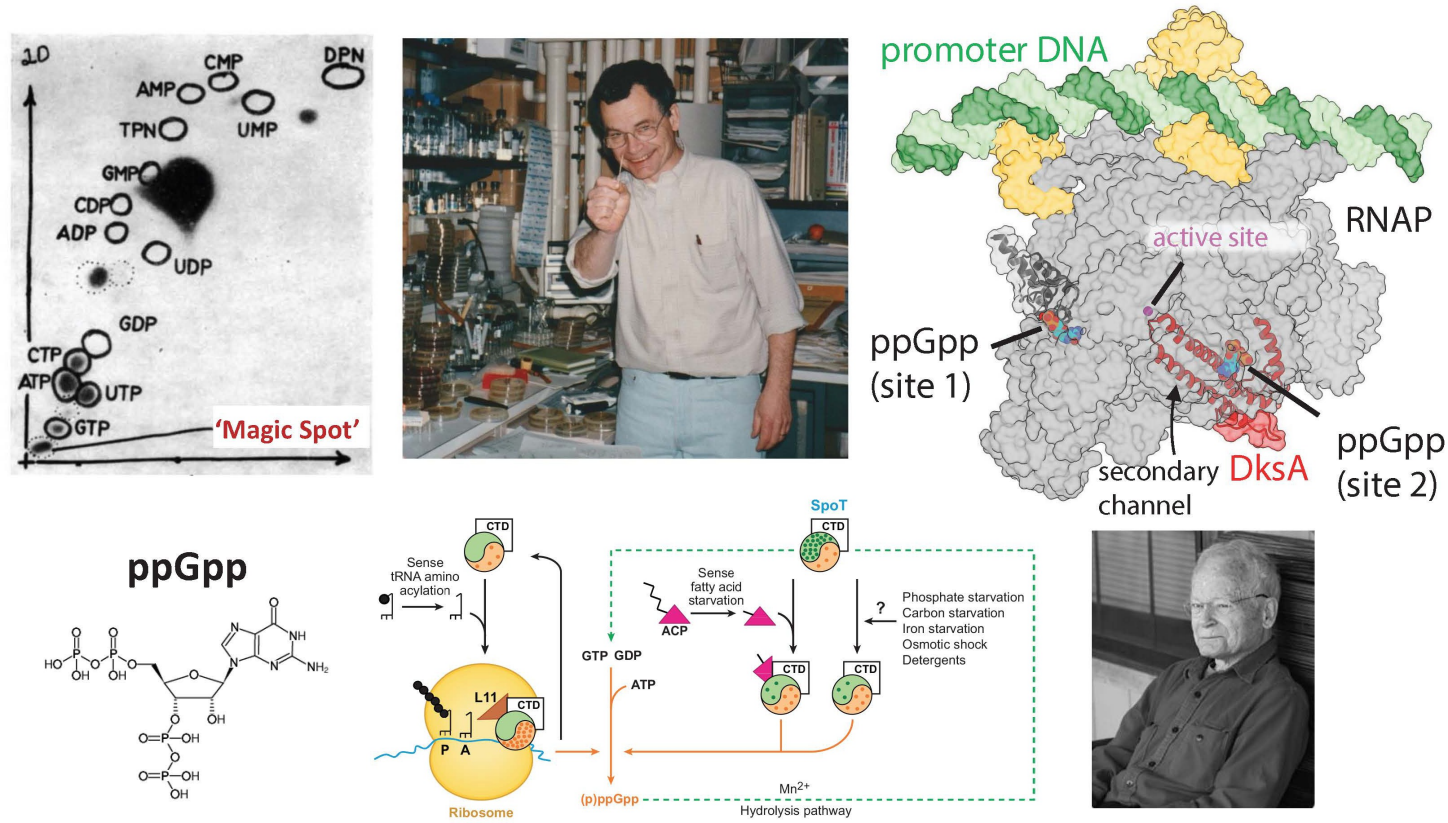
Mike Cashel and (p)ppGpp. Clockwise from top left: Thin layer chromatography showing position of “magic spot,” (p)ppGpp, observed after cells are starved of an amino acid (1); Mike in his lab at NIH; Structure of *E. coli* RNA polymerase (RNAP) showing the two binding sites for (p)ppGpp (contributed by K. Murakami); Mike relaxing at a family reunion; schematic of (p)ppGpp regulation in *E. coli* (2); structure of ppGpp.

After graduating from Amherst, Mike received his MD at Case Western Reserve University. He then joined the Public Health Service at the National Institutes of Health in 1963, working with Ernst Freese. On leave from the NIH, Mike pursued his PhD at the University of Washington with Jonathan Gallant, focusing on the regulatory response termed “stringent control,” the decrease in ribosomal RNA (rRNA) synthesis when cells are starved for an amino acid. The identification of a “relaxed” mutant of *Escherichia coli*, in which rRNA accumulation is no longer perturbed by amino acid starvation, allowed Mike to directly test a model for this control: that the product of the relaxed control mutant generates a phosphorylated metabolite that leads to the observed effects (Fig. 1). Using thin-layer chromatography, he identified two such metabolites, “magic spots,” visible only upon amino acid starvation in the stringent strain but not in the isogenic relaxed mutant. He proposed that these small molecules arose from the sensing of uncharged transfer RNAs (tRNAs) and were likely derivatives of GTP (1) (Fig. 1). Later work soon demonstrated that these were in fact pppGpp (guanosine pentaphosphate) and ppGpp (guanosine tetraphosphate) [referred to collectively as (p)ppGpp] and that they were synthesized under amino acid starvation by the product of the RelA (for relaxed) enzyme. This protein, whose gene was the site of the original relaxed (*rel*) mutants, senses uncharged tRNAs directly by associating with idling ribosomes.

After starting his own lab at the NIH, Mike and colleagues focused their attention on the biology and function(s) of (p)ppGpp. They demonstrated that the addition of (p)ppGpp to an *E. coli* cell-free system reduces the level of rRNA and postulated that the interaction of (p)ppGpp with RNA polymerase selectively downregulates transcription of rRNA, explaining why protein synthesis stops when amino acid levels plummet (3). Later work by other groups, some of whom were mentored by Mike, confirmed the direct binding of (p)ppGpp to RNA polymerase (4–6). Mike and colleagues also determined how (p)ppGpp is synthesized and degraded and how (p)ppGpp works to decrease DNA damage during starvation. Thus, (p)ppGpp is an alarmone (a name Mike did not particularly like), a small molecule acting as a warning system for *E. coli*, to protect the cell’s resources and DNA. In 2013, in a collaboration with the laboratory of Katsu Murakami (Pennsylvania State University), Mike and coworkers revealed one of the first structures of (p)ppGpp bound to RNA polymerase (7). The structure suggested that the binding of (p)ppGpp to an outer pocket of RNA polymerase, created by the *ω* and *β*′ subunits and ~30 Å from the catalytic pocket, allosterically affects the active site for RNA synthesis.

Three years after the initial 2013 structure, crosslinking by the Gourse lab (University of Wisconsin-Madison) identified a second binding site for (p)ppGpp (8). In this case, (p)ppGpp was found together with the protein DksA at the secondary channel of RNA polymerase, the channel where rNTPs enter to travel to the active site. Finally, structures of *E. coli* RNA polymerase ± (p)ppGpp and DksA were published in 2018 by Mike, Katsu, and their colleagues, revealing that the binding of (p)ppGpp to DksA repositions the tip of the DksA protein within the secondary channel (Fig. 1) (9). The repositioning destabilizes the open transcription complex of RNA polymerase and the unwound promoter DNA. Some promoters, like those for rRNA, have unusually unstable open complexes. Thus, (p)ppGpp/DksA is particularly effective at inhibiting rRNA transcription. These structures provided the molecular basis for (p)ppGpp function. In addition, understanding that RNA polymerase has two (p)ppGpp sites that can differentially affect its function unified decades of seemingly contradictory results showing disparate effects of (p)ppGpp and DksA under different conditions.

Ever the pioneer, Mike also explored (p)ppGpp homologs as potential global regulators (10). His efforts to understand candidates and their substrate specificity, including enzymatic sources of synthesis and degradation, led to the development of assays and techniques that will continue to benefit others as they unravel the complex physiological roles of (p)ppNpps (11).

Mike was not a frequent attendee at scientific conferences. However, he was known throughout the scientific community as someone who was always generous with advice, discussion, and sharing strains. As the following edited comments from trainees and colleagues show, Mike’s hospitality and willingness to engage kindly with everyone were traits that all remember first.

Dr. Katsu Murakami: “I first met Mike in 1996 as a graduate student visiting NIH. I knew little of his pioneering work on (p)ppGpp. During the chat over a barbecue, his kindness stood out—he listened to my research ideas and encouraged me to continue transcription research. Working with Mike was always memorable. While reviewers asked for concise revisions of our manuscript, he preferred to deepen our discussions, driven by his relentless curiosity about (p)ppGpp. That passion influenced my own approach to science, teaching me to dig deeper. I remember our long conversations when I stayed at his home for a seminar at the NIH’s Lambda Lunch, and how he showed me that fully understanding a scientific question is always worth the effort. In our last conversation when I visited his house last fall, I promised to finish unraveling the full mechanism of (p)ppGpp-dependent transcription regulation. I wish I could share those final conclusions with Mike and hear his insightful comments once more, even though I know we cannot meet again.”

Dr. Andrew Travers (MRC Laboratory of Molecular Biology) first met Mike in 1970 and continued to interact with him over many decades, although only publishing together once: “He had invited me to give a seminar at NIH. In addition to the science, it was a most enjoyable visit, not without its surprises. Mike was the perfect host to me and my late wife. He lent us his venerable Willys with a round bonnet but unfortunately had neglected to tell us that it had a brake problem—an interesting experience at a busy Washington crossroads. I remember he took us to the Washington Zoo to see the white tigers. On a subsequent visit, he took me to see the azaleas at the National Arboretum, and then, because Mike was so relaxed, we were not allowed to enter a restaurant in central D.C.—we were not wearing ties! The impact of Mike’s science on me has been profound and long-lasting. That I am still studying how (p)ppGpp works 55 years on speaks for itself. What I have learned is that the action of (p)ppGpp (binding at both sites) on RNA polymerase informs on the protein and DNA topology of polymerase assemblies as well as adding to the understanding of the biophysics of the polypeptide chain. One of Mike’s many important contributions was the isolation of multiple “stringent” mutants in *E. coli* mimicking effects of *rel* mutants. I visited Mike in about 2006, and he showed me the pattern of the mutant locations. He did not understand them then, and I could not enlighten him. From what we know now, I would argue that these mutations likely provide a comprehensive model for the molecular basis of the stringent response. Mike was, in essence, a microbiologist, and his work on both growth rate control during exponential growth and also on the mechanisms of the transcriptional switches during the *E. coli* growth cycle provided very fundamental observations. To me, both Mike’s achievements and vision are substantially understated in the literature. The discovery of (p)ppGpp in bacteria is of comparable scientific importance to the discovery of the role of cAMP in eukaryotic systems. It was a great privilege to visit him in March this year (2025) and to see that he was as sharp as ever.”

Dr. Tamara James (Indian Health Service) was a postdoc with Mike at NIH: “I was fortunate to train with Dr. Cashel (Mike) as a postdoctoral fellow where he generously shared his expertise in science, arts, sailing, gardening, and more. He genuinely cared about the success of his mentees. Next to his lab, Mike preferred to work in what NPR might call “Tiny Desk Research” full of papers, instruments, and strains all within arm's reach. It was such an honor to train in a research environment where curiosity was fostered through friendly, sophisticated conversation and gritty approaches that made evident to trainees the rewards of confidence and respect earned through a humble and persistent enthusiasm to learn. He will be missed.’

Dr. Katarzyna Potrykus (University of Gdańsk) was also a postdoc with Mike, who continued their scientific collaboration after she had her own lab: “Working with Mike was a joy and adventure. He allowed his postdocs a lot of freedom to explore different research questions, which gave us the possibility to explore our scientific curiosities. Mike was the most knowledgeable and at the same time the most modest and kindest person I’ve known. I feel very fortunate I got to know him. It was a great privilege to have him as a mentor and a friend.”

Dr. Rajendran Harinarayan (Centre for DNA Fingerprinting and Diagnostics, Hyderabad), another postdoc: “To put it succinctly, Mike saw his lab like an extended family. I experienced this from the day I landed. He received me at the airport, hosted me for a couple of weeks at his house until I found my own accommodation! The lab was invited to numerous get-togethers at his house. Two weekly events open to lab members were a Wednesday lunch outing to a local restaurant (that changed every week) and Friday evening at the Flanagan’s pub in Bethesda (called Friday Seminar) where his scientist friends from NIH or visiting NIH joined. There was a discussion on science and a wide range of topics. While I was focused on working for publications and thinking about a career, I believe he wanted me to appreciate there was more than that to life. I certainly feel fortunate to have had the opportunity to spend time in Mike’s lab.”

The complexity of ways in which (p)ppGpp is made, destroyed, and sensed by RNA polymerase has made this a challenging field for those of us who encounter the tracks of magic spot in our own work. The physiological effects of changing (p)ppGpp levels go well beyond the complex interaction with RNA polymerase and the downstream resulting transcriptional changes; (p)ppGpp directly interacts with and regulates the activity of proteins involved in central metabolism, ribosome assembly, and other processes (12–14). Luckily, Mike Cashel was always happy to consult, offer advice, and mutants. Thus, there are labs all over the world who have depended on his expertise to help them sort through their findings. For me (SG), (p)ppGpp was always too complicated to fully understand, and I never set out to study it, although Mike as well as others had shown that (p)ppGpp null strains did not accumulate the RpoS sigma factor (15), the topic of many of our studies. As many others had found, (p)ppGpp was lurking in our unexplained results. We identified IraP as an anti-adaptor that allowed cells to stabilize and thus accumulate RpoS during phosphate starvation. Unexpectedly, the transcription of *iraP* turned out to be wholly dependent upon positive regulation by (p)ppGpp, explained in part by earlier studies showing that (p)ppGpp increased upon phosphate starvation. With Mike’s critical advice and his mutants in the (p)ppGpp synthesis and degradation machinery, we were able to show that SpoT was responsible for sensing this starvation, and others have since shown that the *iraP* promoter is dependent upon (p)ppGpp *in vitro* (16, 17). This is just one example of how (p)ppGpp has been found to have central roles in regulating cell growth and adaptation to starvation and how the study of (p)ppGpp effects continues to be a subject of intense interest. All of this evolved from Mike Cashel’s initial and continued critical contributions, working at the NIH, digging ever deeper into how (p)ppGpp was made, degraded, and the myriad biological effects it proved to support. His work in *E. coli* has served as the reference point for studies in a wide range of organisms and for multiple “alarmones” in other contexts.

NIH has had a vibrant bacterial and phage research community for many decades, with weekly seminars on a range of topics. Much of what has kept this vibrant has been the attendance and participation of scientists with a range of knowledge and interests, all excited to listen, comment, and argue, as needed. Mike Cashel was an invaluable member of that community—someone who we always wanted to have in the room during a talk—immensely knowledgeable, likely to know a reference the rest of us had not seen or did not remember, and always happy to help us understand the complexities of (p)ppGpp as well as other mysteries of bacterial physiology. Most importantly, Mike always imparted this knowledge with humility and with a contagious passion for gaining deep insight into complicated pathways. He will be sorely missed.

## AFTER EPUB

*[This article was published on 25 February 2026 with an error in Fig. 1. The Figure 1 was corrected in the current version, posted on 3 March 2026.]*
